# Piezoelectric Effect Enhanced Photocatalytic Activity of Pt/Bi_3.4_Gd_0.6_Ti_3_O_12_ Plasmonic Photocatalysis

**DOI:** 10.3390/nano12071170

**Published:** 2022-04-01

**Authors:** Fengjuan Liang, Shijun Wu, Zhiwu Chen, Zhenya Lu

**Affiliations:** School of Materials Science and Engineering, South China University of Technology, Guangzhou 510641, China; 202021021438@mail.scut.edu.cn (F.L.); zhylu@scut.edu.cn (Z.L.)

**Keywords:** bismuth titanate, Pt nanoparticles, heterojunction, piezoelectric effect, plasmonic effect

## Abstract

Novel Pt/Bi_3.4_Gd_0.6_Ti_3_O_12_ heterojunction was synthesized by a decoration of Pt nanoparticles (PtNPs) on the surface of piezoelectric Bi_3.4_Gd_0.6_Ti_3_O_12_ (BGTO) through an impregnation process. The photocatalytic, piezo-catalytic, and piezo-photocatalytic activities of the Pt/BGTO heterojunction for methyl orange (MO) degradation were investigated under ultrasonic excitation and whole spectrum light irradiation. The internal piezoelectric field of BGTO and a plasmonic effect have been proven important for the photocatalytic activity of the heterojunctions. Pt/BGTO exhibited an optimum photocatalytic degradation performance of 92% for MO in 70 min under irradiation of whole light spectrum and ultrasonic coexcitation, and this value was about 1.41 times higher than the degradation rate under whole spectrum light irradiation alone. The PtNPs in Pt/BGTO heterojunction can absorb the incident light intensively, and induce the collective oscillation of surface electrons due to the surface plasmon resonance (SPR) effect, thus generating “hot” electron–hole pairs. The internal piezoelectric field produced in BGTO by ultrasonic can promote the separation of SPR-induced “hot” charge carriers and facilitate the production of highly reactive oxidation radicals, thus enhancing Pt/BGTO heterojunction′s photocatalytic activity for oxidizing organic dyes.

## 1. Introduction

Industrial chemicals and agricultural fertilizers are the major sources of organic contaminants in the effluents which have resulted in grave issues for the environment and the health of living beings [1,2,3]. However, the removal of the contaminants is a challenging task because of their aqueous solubility and stable chemical nature [4,5]. In this regard, there has been a surge of research activities on the application of semiconductor-based photocatalysts as a source of renewable energy and mitigation of aqueous pollutants due to their environment-friendly nature and cost effectiveness [6,7,8,9]. In spite of the significant progress being made in the domain of photocatalysis, practical applications of these materials are hindered because of some thorny issues, including recombination of photogenerated carriers, unresponsive nature in dark conditions, and less efficiency in the solar energy consumption [10,11,12], thus making it imperative to look for new catalysts.

Over the last few years, the piezoelectric polarization-based method known as piezocatalysis has undergone rapid improvement in the domain of environmental mitigation and production of fuels [13,14,15,16]. On the application of mechanical force, the polarization of a piezoelectric semiconductor takes place, leading to the accumulation of oppositely charged localized polarizations on the opposite surfaces. Consequently, the free charge carriers (electrons and holes) are separated after being driven through the built-in piezoelectric field, thereby allowing their participation in the surface redox reactions [17,18,19]. Since the discovery of piezo-electrochemical effect for the first time in 2010 by Hong et al. [20], different piezoelectric materials, including ZnO [21], BaTiO_3_ [22,23], Pb (Zr_0.52_Ti_0.48_)O_3_ (PZT) [24,25], and BiFeO_3_ [26], along with nanocomposites, such as ZnO/TiO_2_ [27] and BaTiO_3_/TiO_2_ [28], have been explored. Nevertheless, most piezoelectric materials show weak conduction of electricity, thus severely restricting charge carrier (or excited electron–hole pair) transport within itself or even across the formed interface (or contact) with other active materials. Additionally, in some reports, piezoelectric heterostructures have been fabricated to utilize the photocatalysis effect generated by the piezotronic or piezophototronic effect for the degradation of dyes and killing of bacteria [29,30,31,32]. Still, enhancement of the photocatalysis efficiency of the piezotronic catalysts via the formation of heterostructure is of great importance.

Recently, plasmonic photocatalysts with high photocatalytic performance, as a consequence of their characteristic localized surface plasmon resonance (LSPR) effect, have been receiving increased attention [33,34,35,36]. The optical response of the nanoparticles consisting of noble metals is the origin of LSPR, which enhances the photocatalytic effect under solar irradiation [37,38,39]. Among the noble metals, Pt finds the widest application as a medium for the LSPR effect in plasmonic photocatalysts due to its cost effectiveness and large optical window in the visible region [40,41,42]. Bi_4_Ti_3_O_12_ with a layered perovskite structure is a piezoelectric material and has been reported to achieve photocatalytic degradation of dyes [43] and hydrogen production via photolysis of water [44]. In addition, the gadolinium-doped Bi_4_Ti_3_O_12_ (Bi_3.4_Gd_0.6_Ti_3_O_12_) has higher ferroelectricity than the pure Bi_4_Ti_3_O_12_ [45]. The improved ferroelectricity is mainly due to the rotation of TiO_6_ octahedra in the a–b plane accompanied by a shift of the octahedron along the a-axis, which is largely enhanced by the substitution element such as Gd, La, and Nd for Bi in the pseudoperovskite [45,46,47]. With this background, our aim was to prepare a bifunctional material by allowing Pt nanoparticles to grow on nanocrystals of Bi_3.4_Gd_0.6_Ti_3_O_12_ in order to combine the phenomena of piezoelectricity with plasmonic photocatalysis. It was hoped to achieve high photocatalytic activity of the piezo-plasmonic photocatalyst based on Pt/Bi_3.4_Gd_0.6_Ti_3_O_12_.

Herein, we enhanced the plasmonic photocatalytic behavior due to the introduction of the piezotronic effect via fabrication of Pt/Bi_3.4_Gd_0.6_Ti_3_O_12_ heterostructure. Due to the polarization effect of Bi_3.4_Gd_0.6_Ti_3_O_12_ nanocrystals generated via sonication induced piezoelectric effect, the recombination of photogenerated hot electron–hole pairs generated on Pt nanoparticles (PtNPs) from LSPR is suppressed. This enhances the photocatalytic degradation of methyl orange (MO) by providing more radicals. The synthesis of photocatalytic Pt/Bi_3.4_Gd_0.6_Ti_3_O_12_ heterostructure was carried out employing an impregnation method. Investigations were carried out to understand the effect of Pt loading and optical behavior of the heterojunction on the photocatalytic disintegration of dye. The photocatalytic behavior of Pt/Bi_3.4_Gd_0.6_Ti_3_O_12_ heterostructure indicates that under the presence of the entire solar spectrum together with ultrasonic excitation, 92% MO was degraded within 70 min and was 1.41 times higher than the degradation rate under light excitation alone. The mechanism behind the enhanced piezo-photocatalytic effect was explained based on the surface-enhanced Raman scattering (SERS) test and analysis of the energy band diagram. The photocatalytic activity of Pt/Bi_3.4_Gd_0.6_Ti_3_O_12_ heterostructure, i.e., piezo-photocatalysis, is generated by the synergistic combination of plasmonic photocatalysis (PtNPs) and piezoelectric effects (Bi_3.4_Gd_0.6_Ti_3_O_12_). Current work offers an easy, cost-effective means for the preparation of photocatalysts with enhanced performance having immense potential in the remediation of environmental pollution, which can also be extended to other piezoelectric systems.

## 2. Experimental Method

### 2.1. Synthesis of BGTO and Pt/BGTO Samples

**Figure 1:**
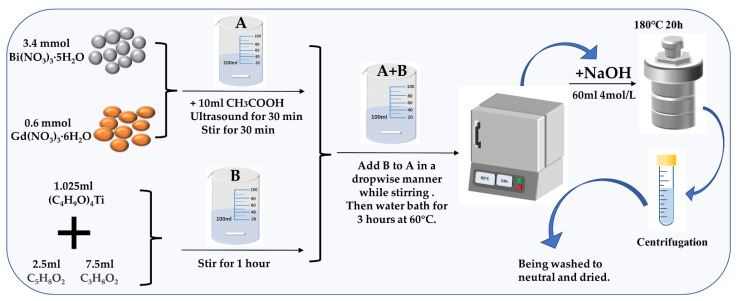
Schematic representation of the preparation procedure for BGTO.

Bi_3.4_Gd_0.6_Ti_3_O_12_ (BGTO) powders were obtained via sol–gel hydrothermal technique using analytical grade bismuth nitrate pentahydrate (Bi(NO_3_)_3_·5H_2_O), gadolinium nitrate hexahydrate (Gd(NO_3_)_3_·6H_2_O), tetrabutyl titanate (Ti(C_4_H_9_O)_4_), acetic acid (CH_3_COOH), acetylacetone (C_5_H_8_O_2_), and ethylene glycol monomethylether (C_3_H_8_O_2_). First, according to the composition of Bi_3.4_Gd_0.6_Ti_3_O_12_, Bi(NO_3_)_3_·5H_2_O (3.4 mmol) and Gd(NO_3_)_3_·6H_2_O with the corresponding ratio were placed in a 100 mL beaker, followed by the addition of 10 mL CH_3_COOH as a solvent for completely transforming the mixture into solution (A) under magnetic stirring (400 rpm) for 1 h at the ambient temperature. Then, a stoichiometric amount of Ti(C_4_H_9_O)_4_ was slowly added into the mixture of 5 mL C_5_H_8_O_2_ and 10 mL C_3_H_8_O_2_ dropwise for complete mixing under magnetic stirring (400 rpm) for 1 h to form solution B. Subsequently solution B and solution A were mixed under vigorous stirring (600 rpm) over 1 h resulting in a homogeneous sol, which was then converted into a dry gel by treating it over 24 h in the presence of 85 °C in a drying oven. A suspension of the dry gel was made by adding 70 mL 4M NaOH solution, which was then shifted into a Teflon-lined SS autoclave having a volume of 100 mL. Thereafter, the heating of the autoclave was continued for 24 h at a temperature of 180 °C. Finally, the obtained BGTO product was centrifuged at 8000 rpm for 30 min and washed three times with DI water, and then dried for 24 h at 100 °C in a drying oven. Figure 1 shows the preparation procedure for BGTO.

The Pt/BGTO was synthesized via an impregnation route. The as-prepared BGTO powders were dispersed in an ethanol solution with a predetermined weight of H_2_PtCl_6_·6H_2_O (3 wt% loading). Subsequently, stirring (600 rpm) was continued for 6 h under normal pressure for uniform deposition of H_2_PtCl_6_ on the surface of BGTO, which was then dried by using a rotary evaporator. Finally, after overnight drying at 85 °C, all of the product were heat treated for 2 h at 450 °C inside a tube furnace in the presence of N_2_/H_2_ (5 vol% H_2_) gas flow. The gas flow rate was 100 mL·min^−1^.

### 2.2. Samples Characterization

The phase structures of samples were recorded by X-ray powder diffraction (XRD; Rigaku D/Max-3C, Tokyo, Japan). A Zeiss Merlin field-emission scanning electron microscope (SEM, Oberkochen, Germany) was used to characterize the morphology of the samples. Regular and high-resolution transmission electron microscopies (HRTEM) were conducted using a JEOL JEM 2100F (Tokyo, Japan) instrument. Raman spectroscopy was performed at room temperature with a Raman spectrometer (HJY LabRAM Aramis, Paris, France) at an excitation wavelength of 473 nm and a power of 22 mW. The UV-Vis diffuse reflection spectra (DRS) of the samples were recorded on a UV-Vis spectrophotometer (Shimadzu, UV-2550, Kyoto, Japan). X-ray photoelectron spectroscopy (XPS) was conducted using Perkin Elmer GX instruments (Waltham, MA, USA). A PerkinElmer LS55 spectrofluorometer (Waltham, MA, USA) was used to characterize the photoluminescence (PL) spectra of the samples. 

The photocurrent measurement was carried out on a CHI-660E electrochemical analyzer (Chenhua, Shanghai, China) using a three-electrode cell system with indium tin oxide (ITO/BGTO (or Pt/BGTO) as the working electrode, platinum wire as the counter electrode, and standard calomel electrode (SCE) as the reference. A 300 W Xe lamp with a cutoff filter of 420 nm was utilized as the light source. Furthermore, the (ITO/BGTO (or Pt/BGTO) electrodes were fabricated as follows: first, BGTO (or Pt/BGTO) samples (5 mg) were added into solutions containing ethanol (0.15 mL) and 5% Nafion D-520 (0.35 mL) and ultrasonicated for 20 min. Then, the resultant BGTO (or Pt/BGTO) slurry (0.1 mL) was sintered at 100 °C for 2 h after being cast onto precleaned ITO glass. 

Piezo-response force microscopy (PFM) was carried out using an Asylum Research Cypher ES atomic force microscope equipped with a Pt-coated Si cantilever (TipsNano, NanoWorld Arrow-EFM) with a resonance frequency of ~75 kHz and a spring constant of ~2.8 N m^−1^. A train of DC pulses with a constant duration of 20 ms and amplitude from −10 V to 10 V and back to −10 V was applied to the cantilever tip staying at a fixed position on the surface of the BGTO (or Pt/BGTO) sample.

### 2.3. Catalytic Activity Assessment

**Figure 2:**
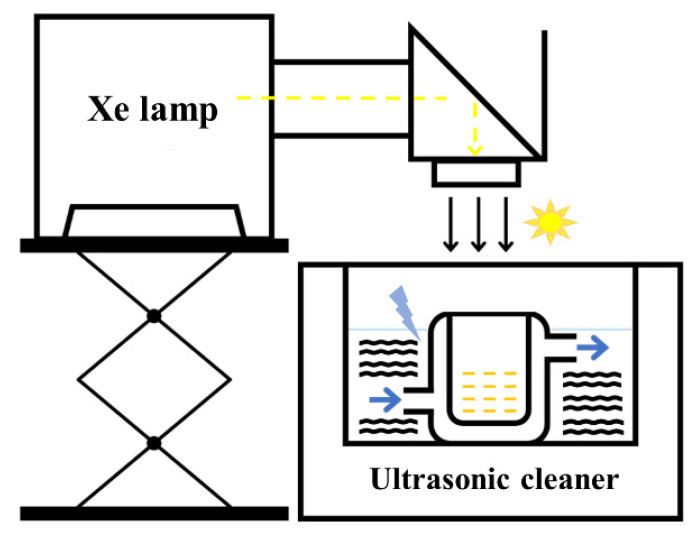
Schematic illustration of an ultrasonic/photoreaction device.

The photocatalytic, piezo-catalytic, and piezo-photocatalytic activity of the BGTO (or Pt/BGTO) heterojunctions toward MO degradation were investigated under ultrasonic (53 kHz, 100 W) and whole spectrum light irradiation excitation. A total of 80 mL solution of MO (10 mg·L^−1^) and 20 mg photocatalyst were placed in a 150 mL beaker. Prior to irradiation, the suspensions were continuously stirred in the dark for 30 min to reach adsorption–desorption equilibrium. In order to avoid the influence of the pyro-catalytic effect, the temperature fluctuation of the suspension was less than 1 °C, controlled by a circulating cooling water system. During photocatalysis and piezo-photocatalysis, a 300 W Xenon lamp (PLS-SXE300D, Beijing Perfectlight, Beijing, China) without a cutoff filter was used as a light source, which gave an irradiation intensity of 100 mW·cm^−2^. A total of 4 mL solution was obtained for sampling at fixed intervals, followed by centrifugation to remove any catalyst powder. The absorption spectra of the MO solution were recorded by UV-Vis spectroscopic tests (Yoke UV1901PC, Shanghai, China). The schematic illustration of an ultrasonic/photoreaction device is shown in Figure 2.

## 3. Results and Discussion

**Figure 3:**
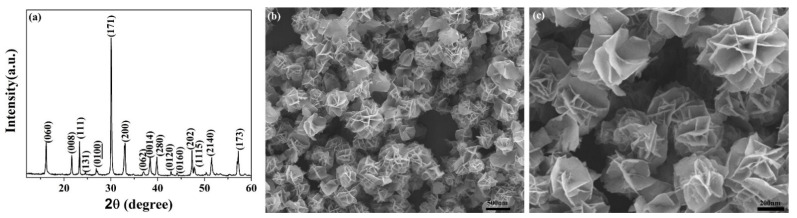
(**a**) XRD pattern, (**b**) low and (**c**) high magnification SEM images of synthesized BGTO powders.

XRD peaks of the synthesized BGTO powders are shown in Figure 3a. The synthesized BGTO showed characteristic peaks corresponding to Bi_4_Ti_3_O_12_ which belong to the space group Fmmm and the structure was orthorhombic (JCPDS No.73-2181). The absence of additional peaks indicated the single-crystalline nature of the photocatalyst. The sample with a single phase had good crystallinity, as indicated by the sharpness of the obtained XRD peaks. Even after the addition of Gd, the signature peaks of the Bi_4_Ti_3_O_12_ phase remained undisturbed, thus indicating the formation of the solid solution via diffusion of Gd inside the lattice of perovskite. Figure 3b shows a representative SEM image of the as-prepared BGTO, which shows the unambiguous presence of flower-like superstructures. The assembly of multiple two-dimensional nanoplates was responsible for the formation of flower-like superstructures, as can be seen in Figure 3c. The average diameter of BGTO microflowers was about 430 nm, while the average thickness of nanoplates was about 10 nm. In addition, the TEM image of the BGTO (Figure S1) also shows that the multiple nanoplates are assembled into a flower-like microstructure, in line with the SEM results.

In this study, surface deposition of PtNPs took place on BGTO via an impregnation process. When the as-prepared BGTO sample was deposited with PtNPs, its color turned gray, but its XRD pattern (Figure S2a) and SEM image (Figure S2b) showed no evident change, probably because the amounts of deposited PtNPs were extremely minuscule to be detected [48,49]. 

**Figure 4:**
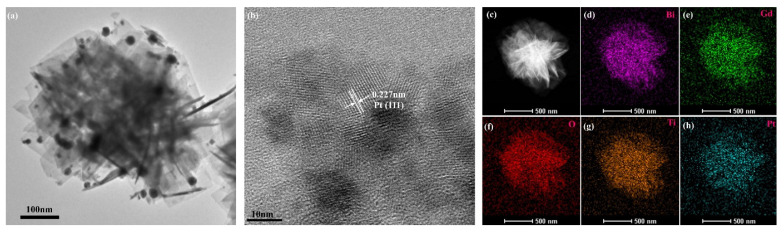
(**a**) TEM image, (**b**) HRTEM image of Pt/BGTO, (**c**–**h**) STEM image, and EDX elemental mapping images of the Pt/BGTO heterojunction.

Loading of PtNPs on the BGTO was confirmed by TEM and XPS analysis. As can be seen in Figure 4a, PtNPs with an average diameter of 11 nm were dispersed on the surface of BGTO transparent nanosheets. HRTEM image of Pt/BGTO exhibits distinct lattice fringes of 0.227 nm (Figure 4b), agreeing well with the d-spacing of the (111) planes of Pt^0^. Figure 4c–h show STEM image and EDX elemental mapping images of the Pt/BGTO heterojunction, which exhibited the homogeneous distribution of Bi, Gd, O, Ti, and Pt, further verifying that the PtNPs are uniformly deposited on the BGTO surface. The EDS spectrum analysis of Pt/BGTO (Figure S3) also showed that all the peaks can be ascribed to Bi, Gd, Ti, O, and Pt elements. As shown in Table S1, the molar ratio was estimated to be about 17.69: 3.12: 15.59: 62.71: 0.89, very close to the theoretically calculated ratio. In addition, the weight percentage of Pt obtained from the EDS analysis in Pt/BGTO was 2.73 wt% (the theoretical value was 3.0 wt%).

**Figure 5:**
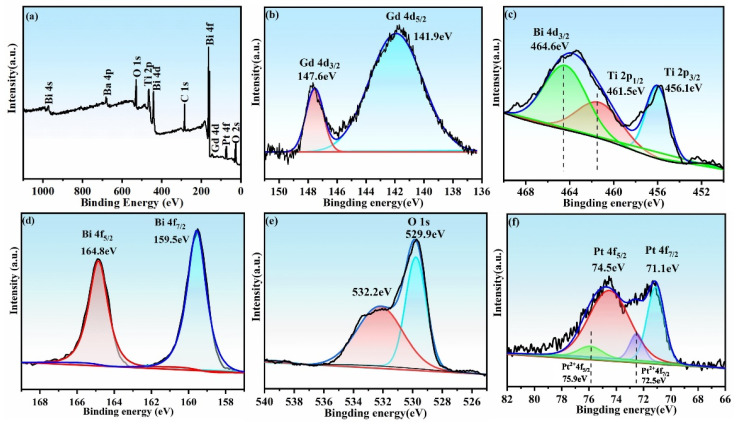
(**a**) XPS survey spectrum of Pt/BGTO heterojunction. High reolution XPS spectra of (**b**) Gd 4d, (**c**) Ti 2p, (**d**) Bi 4f, (**e**) O 1s, and (**f**) Pt 4f in Pt/BGTO heterojunction.

XPS technique was employed for investigating the chemical environment of elements Pt, Bi, Gd, Ti, and O in the Pt/BGTO heterojunction. The survey XPS spectra (Figure 5a) revealed that all the peaks can be ascribed to Pt, Bi, Gd, Ti, O, and C (carbonaceous compounds in the instrument). High-resolution XPS spectra of Gd 4d, Ti 2P, Bi 4f, O 1s, and Pt 4f levels and their deconvoluted plots can be seen in Figure 5b–f. The two peaks at around 141.9 eV (Gd 4d 5/2) and 147.6 (Gd 4d 3/2) seen in the Gd 4d spectrum, as displayed in Figure 5b, could be due to trivalent gadolinium (Gd^3+^) [41]. For Ti, a broad bump is observed at around 464 eV, which is due to the partial overlapping of Ti 2p1/2 and Bi 4d3/2 [50]. According to the peak deconvolution, the two peaks at around 456.1 and 461.5 eV in Figure 5c could be correlated with Ti 2p 3/2 and Ti 2p 1/2 states of Ti^4+^, respectively, whereas the 464.6 eV signal corresponds to the Bi 4d3/2 energy state [50]. As observed from the deconvoluted Bi 4f spectrum shown in Figure 5d, the presence of two main peaks centered at around 159.5 and 164.8 eV represents the binding energies of Bi 4f 7/2 and Bi 4f 5/2, respectively, thus confirming Bi^3+^ to be the species of Bi [51,52]. The wide and asymmetric nature of the O 1s spectra in (Figure 5e) points toward the presence of multiple chemical states for oxygen on the nanosheet surface. When deconvoluting the 1s spectrum of O, the contribution from two different oxygen-bearing species was observed. The peak at a lower binding energy of 529.9 eV corresponds to lattice oxygen (O^2−^ in the stronger Ti–O bond), while the peak at higher binding energy (532.2 eV) is associated with the surface adsorbed oxygen (–OH group and chemisorbed oxygen-containing species) [53]. As shown in Figure 5f, the Pt 4f signal can be fitted into four symmetric peaks. The respective peaks at 71.5 eV and 74.6 eV are due to the Pt 4f 7/2 and Pt 4f 5/2 species of Pt^0^, respectively, whereas the other two peaks at around 72.5 and 75.9 eV could be correlated with Pt 4f 7/2 and Pt 4f 5/2 states of Pt^2+^, respectively [54,55]. On the basis of the relative XPS areas, the atomic percentages of Pt^0^ and Pt^2+^ in the sample are around 90.9% and 9.1%, respectively, confirming that the loaded Pt is mainly present in the metallic state in Pt/BGTO heterostructure. The existence of a small amount of Pt^2+^ species may be attributed to the formation of a Pt–O bond caused by the oxygen chemisorption on the PtNPs surface. The atomic composition percentages of Bi, Gd, Ti, O, and Pt retrieved by XPS for Pt/BGTO were shown in Table S2, which were close to the theoretically calculated ratio. The results obtained from XPS further confirmed that Pt/BGTO heterojunctions were synthesized successfully. 

**Figure 6:**
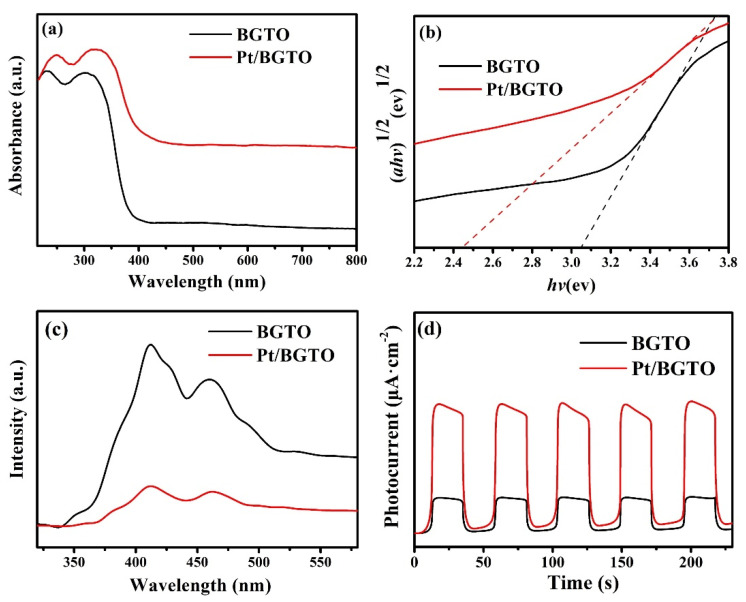
(**a**) UV−vis DRS and (**b**) plot of the transformed Kubelka−Munk function [F(R∞)] versus the photon energy (*hν*) for the BGTO and Pt/BGTO samples; (**c**) PL spectra and (**d**) Photocurrent response of the BGTO and Pt/BGTO samples.

The optical absorption behavior of a photocatalyst is a major parameter in deciding its photocatalytic properties. The UV-vis DRS spectra of pure BGTO and Pt/BGTO heterojunction are shown in Figure 6a. Pt is able to change the light-harvesting capability of BGTO. The absorption edge of pure BGTO was observed at 380 nm. In a striking difference from the pure BGTO, Pt/BGTO showed a strong ability to absorb visible light of the spectrum (up to 700 nm) and could be attributed to the surface plasmon resonance (SPR) of PtNPs [56,57]. A plot of (*ahv*)^1/2^ versus *hv* could be utilized for obtaining the absorption bandgap values via extrapolation of its tangent line to the energy axis at *a* = 0, and the obtained bandgap values were found to be 3.05 and 2.45 for pure BGTO and Pt/BGTO, respectively (Figure 6b). Thus, Pt was able to reduce the absorption bandgap of BGTO and consequently increased its capacity to absorb light. The phenomena can be explained based on the following discussion. First, the SPR effect of the PtNPs was responsible for the absorption of visible light. In this, oscillation and resonance of hot electrons present on the surface of PtNPs with the incident light resulted in an increase of electromagnetic field in the vicinity of the Pt surface, which prompted the absorption of visible light by Pt/BGTO heterojunctions [56,57]. Second, there is the formation of the Schottky barrier at the interface between PtNPs and a semiconductor of the heterostructure, which creates some lower energy levels, thus changing the original energy equilibrium [58]. This facilitates the charge-transfer transition between noble metals and semiconductors, as has been described by Sayama et al. [59]. Enhancement of the light absorption behavior of BGTO due to loading of Pt cocatalyst is expected to improve the photocatalytic performance of BGTO, which is described below.

The efficiency of the photocatalytic process is hampered because of the electron–hole recombination, and hence the excitation/separation ability of the charge carriers of BGTO and Pt/BGTO was evaluated via photoluminescence (PL) spectra employing 325 nm as the excitation wavelength (Figure 6c). Reduced intensity for the PL peak implies an increased separation between the electron–hole pairs [60,61]. Pt/BGTO showed distinctly much lower intensity for the PL peak compared with that of BGTO, implying that the photogenerated electron-hole pairs in Pt/BGTO had a lower rate of recombination. Enhanced separation of the photogenerated charge carriers in Pt/BGTO heterostructure presents a higher possibility for the formation of active free radicals (OH·) in the consequent MO degradation, which may potentially enhance the photocatalytic efficiency. Additionally, further enhancement of photocatalytic activity takes place due to improved separation efficiency of the photogenerated carriers as a consequence of the conduction band of BGTO acting as an electron trap. Photocurrent, which is another crucial parameter for photocatalytic reactions, is directly affected by the separation and transfer of photogenerated carriers. The concurrent photocurrents for the samples of BGTO and Pt/BGTO were measured after being illuminated by the whole spectrum. The higher the photocurrent response, the higher is the charge carrier density, and the more efficient is the separation between the charge carriers [61,62], thus making it beneficial for photocatalytic performances. As represented in Figure 6d, considerably higher photocurrent density was found for the Pt/BGTO sample in comparison with that of pure BGTO, demonstrating enhanced charge separation and transfer capability for the Pt/BGTO heterojunction structure, which is favorable towards its photocatalytic performance. The results of the photocurrent test are in agreement with the PL spectra (Figure 6c) and catalytic activity of samples characterized later.

**Figure 7:**
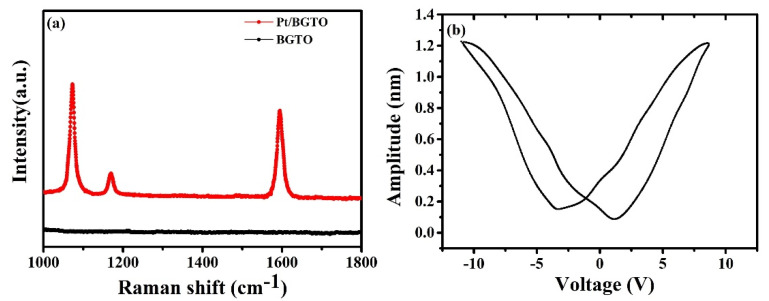
(**a**) SERS intensity profile of 4−MBA on the BGTO and Pt/BGTO samples; (**b**) the amplitude−voltage curves of Pt/BGTO sample.

The enhancement of electromagnetic field near PtNPs was determined via surface-enhanced Raman scattering (SERS) employing 4 mercaptobenzoic acid (4-MBA) as a probe molecule to confirm the presence of localized SPR effect (Figure 7a) [63]. Two strong peaks corresponding to the vibration (ν8a) and breathing (ν12a) modes of an aromatic ring were observed at 1074 and 1594 cm^−1^, respectively, while the weak peak at 1170 cm^−1^ corresponds to the C-H deformation. As for pure BGTO, no peaks could be observed corresponding to 4-MBA. In contrast, the Raman spectrum of the Pt/BGTO heterojunction exhibited all the abovementioned peaks belonging to 4-MBA. A highly sensitive SERS probe based on Pt/BGTO heterojunction further proved the strong SPR effect and electromagnetic field induced by plasmon–exciton interaction from the adherent PtNPs on the heterojunction surface. To further prove that the BGTO has piezoelectric properties, we measured the piezoelectricity of Pt/BGTO heterojunction by PFM. Figure 7b shows the amplitude–voltage curves of Pt/BGTO, which exhibited the typical amplitude–voltage “butterfly loop” curve under the action of an external electric field with a constant amplitude and period, confirming the piezoelectric nature of Pt/BGTO. The maximum displacement was 1.23 nm at a voltage bias of −10.1 V, indicating that a single Pt/BGTO heterojunction has outstanding piezoelectric properties [64,65]. 

**Figure 8:**
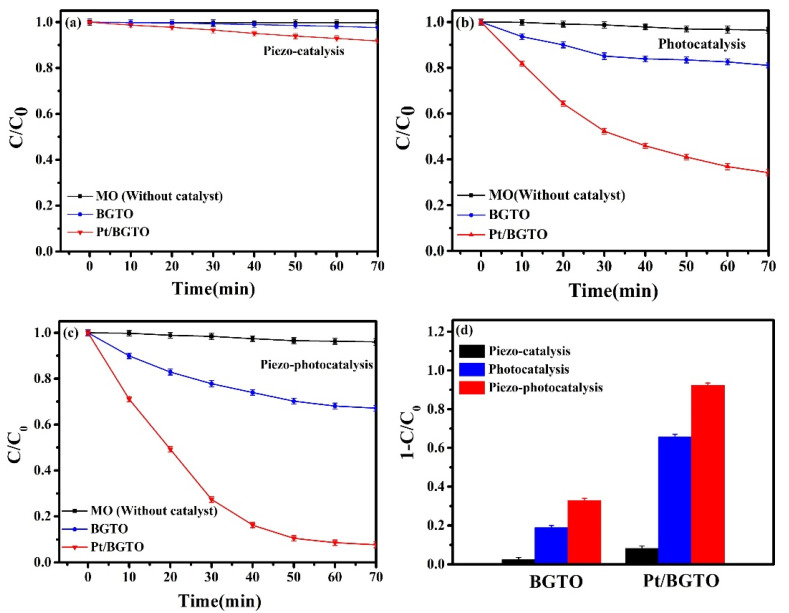
Degradation of MO under (**a**) ultrasonic excitation, (**b**) whole spectrum light irradiation, (**c**) both ultrasonic excitation and whole spectrum light irradiation, (**d**) piezo-catalytic, photocatalytic, and piezo-photocatalytic degradation of MO in the presence of BGTO and Pt/BGTO samples for 70 min.

The degradation of MO was chosen as the experimental subject to elucidate the effect of piezoelectricity on the photocatalytic performance of Pt/BGTO heterojunction, employing irradiation by light of the whole spectrum ultrasonic excitation as well as both together. Ultrasound was used as the excitation source for inducing the piezoelectric field. For the application of ultrasonic excitation alone (Figure 8a) in the absence of light irradiation, less than 10% degradation of MO was observed under the presence of BGTO and Pt/BGTO (Figure 8a). This signifies the limited effect of the polarization charges produced by the built-in electric field in BGTO, which was not involved in the catalytic process. A comparative evaluation of the piezo-catalytic activity for pure BTO and Pt/BGTO is shown in Figure 8a, which shows limited enhancement due to the decoration of BGTO by PtNPs (from 3% to 9%). A whole spectrum of light was used to evaluate the photodegradation of MO over pure BGTO and Pt/BGTO samples (Figure 8b). The weak photocatalytic activity was observed for pure BGTO, with the rate of degradation of MO being less than 20% in 70 min, thus revealing the wide bandgap to be the reason behind the intrinsic poor photocatalytic effect of BGTO. In comparison, dramatic enhancement of photocatalytic activity was observed for Pt/BGTO, with the consequent degradation of MO being more than 65% within 70 min as a result of the LSPR effect, i.e., the PtNPs being a strong absorber of irradiated light, thus inducing the surface electrons to oscillate collectively and produce hot electron–hole pairs, and, through the construction of heterostructures, suppress their recombination. Therefore, key factors in enhancing the formation of OH· and oxidation of MO are the plasmonic effect and formation of the hybrid structure (detailed discussion in the catalyst mechanism section). Distinct enhancement of the degradation rate of MO was observed when the whole spectrum of the light was used for irradiation together with ultrasonic excitation. More than 92% MO was degraded within 70 min for Pt/BGTO, with the rate being 1.41 times faster than the degradation under light irradiation only in the absence of ultrasound excitation. Under the presence of both the excitation means, pure BGTO still showed a low degradation rate (rate of degradation less than 33% in 70 min, Figure 8c). Significant improvement of the photocatalysis performance of Pt/BGTO heterojunction in the presence of both photoexcitation and ultrasonic excitation can be assigned to the evoking of additional piezoelectric polarization of BGTO together with a built-in piezoelectric field due to ultrasonic excitation, which helps to accelerate the separation of hot electron–hole pairs and boost the photocatalytic properties. Moreover, as shown in Figure S4 (Supplementary Material), the degradation rate of MO for Pt/BGTO remained stable after the fourth cycled run under both ultrasonic excitation, and the whole spectrum light irradiation can be maintained above 90.8%, which demonstrates the excellent reusability of the Pt/BGTO photocatalyst. As shown in Table S2, the atomic percentages of various elements retrieved by XPS for Pt/BGTO after photocatalytic reaction (under both ultrasonic excitation and whole spectrum light irradiation) were almost the same as that for pristine Pt/BGTO, indicating the excellent stability of the Pt/BGTO photocatalyst. The MO degradation results for all the samples under the abovementioned conditions are summarized in Figure 8d. The catalytic process is affected minutely by the ultrasonic excitation alone. Pure BGTO showed negligible catalytic performance under ultrasonic excitation (the first black column), thus excluding the influences of extrusion, collision, and vibration of solution molecules induced by ultrasonic excitation on the oxidation of MO molecules. The Pt/BGTO heterojunction showed slight catalytic behavior (other black columns), which can be ascribed to the generation of piezoelectric potential in BGTO in the presence of ultrasonic excitation, leading to the excitement of limited charge carries on PtNPs, which can induce small amounts of free radicals. When we introduced the excitation by whole light spectrum, a slight increase in the photocatalytic performance was observed for the pure BGTO sample, with the MO degradation rate increasing to 19%. For the Pt/BGTO heterojunction, under the presence of light irradiation, more than 65% of MO was observed to be degraded. This can be attributed to the introduction of hydroxyl radicals by the photogenerated charge carriers resulting in the oxidation of organic compounds. Furthermore, for the application of ultrasonic excitation and light irradiation together, Pt/BGTO heterojunction showed more than 92% degradation of MO (pure BGTO sample shows only 33% catalytic degradation). Improvement in MO degradation by ~59% for the Pt/BGTO heterojunction photocatalysts is due to the participation of ultrasonic excitation and the introduction of piezoelectric polarization (equivalent to a built-in electric field) in BGTO. The built-in electric field subsequently enhances the separation and transport of photogenerated electron–hole pairs at the heterostructure interface and promotes free radicals generation. The Pt/BGTO sample demonstrated higher photocatalytic activity compared with other piezo-photocatalysts recently reported under similar conditions (as presented in Table S3).

We have performed a control experiment depositing PtNPs onto the surface of Bi_2_O_3_, an inert support (not piezoelectric). As for both Bi_2_O_3_ and Pt/Bi_2_O_3_ samples, no enhancement of the degradation rate of MO was observed when the photoexcitation and ultrasonic excitation were used together (Figure S5). The result of the control experiment means that the built-in piezoelectric field in BGTO induced by ultrasonic excitation is the key to promoting the mobility of the charge carriers and suppressing the recombination of photogenerated carriers, which subsequently contributes to the photocatalysis process.

**Figure 9:**
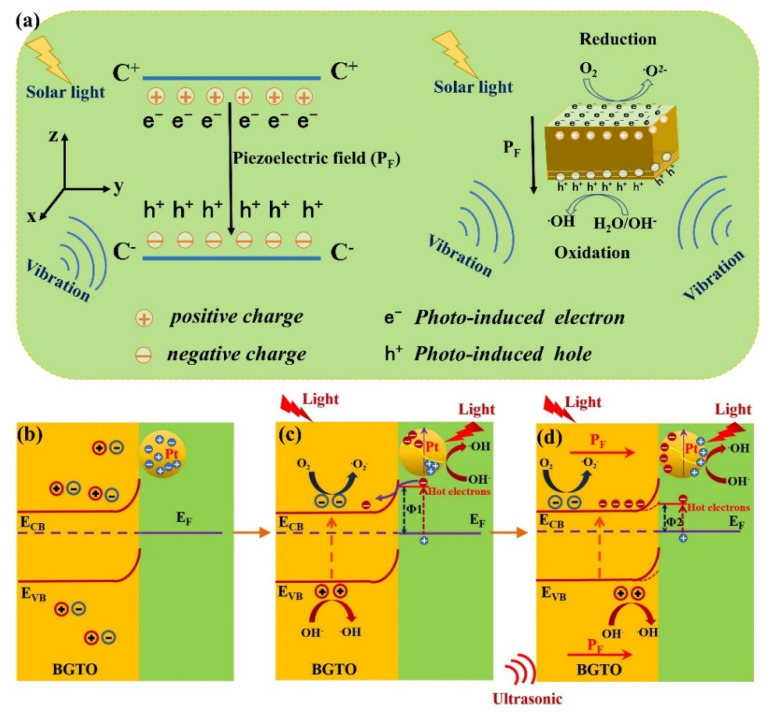
(**a**) Schematic illustration of piezoelectric effect. Schematic illustration of the synergistic piezoelectric effect and exciton−plasmon interaction. (**b**) Schottky barrier between Pt and BGTO; (**c**) Pt/BGTO under whole spectrum light irradiation; (**d**) Pt/BGTO under ultrasonic excitation and whole spectrum light irradiation together.

For the classical piezoelectric materials, such as Pb(Zr,Ti)O_3_ and BaTiO_3_, applied external stress induces the displacement of the charge centers of the cations and anions, creates aligned dipole moments, and results in an inner electric field, i.e., internal piezoelectric field (P_F_). As shown in Figure 9a, the internal piezoelectric field facilitates the separation of photo-induced electrons and holes immediately after their generation, realizing in situ catalytic degradations of pollutants for environmental purification and water splitting for hydrogen generation [22,23,24,25]. BGTO with a layered perovskite structure is a piezoelectric material, and applied external stress (ultrasound is used as the excitation source) can also induce the P_F_ in BGTO crystals, similar to the Pb(Zr,Ti)O_3_ and BaTiO_3_. The superior piezo-photocatalysis performance by Pt/BGTO heterojunction can be elucidated in depth via an energy band diagram. The pure BGTO can be considered as a semiconductor having a large bandgap (bandgap ≈ 3.05 eV). When the PtNPs are decorated on BGTO, the difference in Fermi levels between Pt and BGTO leads to the redistribution of charge carriers at the interface. This results in the formation of a Schottky barrier in the interfacial contact area (Figure 9b). The formed Schottky barrier can potentially prevent the reunification of excited electron hole pairs and force the excited holes toward the PtNP/solution interface [66]. When a Pt/BGTO heterojunction is irradiated by the whole spectrum of light (Figure 9c), BGTO in the Pt/BGTO heterojunction can be excited by light to generate electron–hole pairs, which would be detained by acceptor and donor in the solution, respectively, leading to further formation of active hydroxyl radicals (·OH) and superoxide radicals (·O_2_^−^) for the oxidation of MO. However, the photogenerated electron–hole pairs were very limited as a result of their wide bandgap. Therefore, as shown in Figure 8b, pure BGTO without Pt modification showed weak degradation activity for MO under light irradiation alone. Meanwhile, PtNPs in the Pt/BGTO heterojunction can be excited by incident light to generate an electromagnetic field caused by the SPR effect, which drives the collective oscillation of electrons. The highly sensitive SERS probe based on Pt/BGTO (Figure 7a) proved the strong SPR and electromagnetic field induced by adnexed PtNPs on the heterojunction surface. Tasi et al. showed that SPR could boost the generation of electrons and holes through two different effects—the SPR sensitization effect and the SPR-powered bandgap breaking effect [67]. Brongersma et al. argued that plasmon excitations in metallic nanostructures can be engineered to enhance and provide valuable control over the emission of hot carriers. Plasmon resonances in nanostructures can be damped radiatively by re-emission of a photon or nonradiatively through the creation of hot electron–hole pairs via Landau damping [68]. So, when Pt/BGTO is irradiated by the whole spectrum of light, the surface electrons of the PtNPs exhibit collective oscillations (i.e., SPR) and decay into energetic “hot” electrons. The “hot” electrons get transferred to the BGTO via the Schottky barrier and are subsequently transformed to ·O^2−^ to oxidize MO, whereas the positive holes which are left behind play a role in the generation of ·OH and start MO oxidation. When the Pt/BGTO heterojunction is stimulated by both irradiation of the whole light spectrum and ultrasonic excitation (Figure 9d), the BGTO in Pt/BGTO heterojunction is deformed, and a piezoelectric internal electric field (P_F_) is formed inside due to the piezoelectric effect (Figure 9a). The piezoelectric field in BGTO can decrease the Schottky barrier at the Pt/BGTO heterojunction [69,70,71,72], which assists in the separation of hot electron–hole pairs generated by the LSPR effect and attracting more hot electrons to the BGTO. In addition, the piezoelectric fields in BGTO can also suppress the recombination of electron–hole pairs inside BGTO (excited by incident light). Therefore, more excited holes can migrate to PtNP/solution interface, and more hot electrons can migrate from PtNPs to BGTO, leading to the formation of more radicals for the oxidation of MO, i.e., enhancement of plasmonic photocatalysis took place via a piezoelectric effect in this Pt/BGTO heterojunction.

## 4. Conclusions

In summary, Pt/BGTO plasmonic photocatalyst was successfully synthesized, and a comprehensive evaluation of its photocatalytic activity was carried out in order to investigate the piezo-photocatalysis process. Enhanced plasmonic photocatalysis activity due to efficient piezoelectric effect was realized via coupling of the internal piezoelectric field and plasmonic photocatalytic process. The Pt/BGTO heterojunction exhibited a high photocatalytic degradation performance of 92% for MO in 70 min in the presence of the whole light spectrum along with ultrasonic coexcitation, and this value was ~1.41 times faster than the rate of degradation in the presence of whole light spectrum only. PtNPs in the Pt/BGTO heterojunction can absorb incident light strongly to generate oppositely charged hot pairs owing to the SPR effect. The internal piezoelectric field can separate the photogenerated electrons and holes inside BGTO and decrease the recombination of hot electron–hole pairs in PtNPs due to SPR, forcing more hot electrons to transfer from Pt to BGTO and more excited holes to migrate to the PtNP/solution interface, thus generating more radicals for the oxidation of MO, that is, the piezoelectric effect enhanced plasma photocatalysis. This work demonstrates that piezoelectric plasmonic photocatalysis is an advanced and efficient wastewater purification technology that has great application prospects in the field of environmental remediation.

## Data Availability

The data presented in this study are available in this article.

## Supplementary Materials

The following are available online at https://www.mdpi.com/article/10.3390/nano12071170/s1, Figure S1. TEM image of synthesized BGTO powders, Figure S2: (a) XRD pattern, (b) SEM image of Pt/BGTO, Figure S3: EDS spectrum analysis of the Pt/BGTO, Figure S4: Cycling runs of MO degradation by Pt/BGTO heterojunction under both ultrasonic excitation and whole spectrum light irradiation, Figure S5: Piezo-catalytic, photocatalytic, and piezo-photocatalytic degradation of MO in the presence of Bi_2_O_3_ and Pt/Bi_2_O_3_ samples for 70 min, Table S1: The atomic percentages and weight percentages of Pt Bi, Gd, Ti, O, and Pt obtained from the EDS spectrum analysis in Pt/BGTO, Table S2: The atomic compositions percentages retrieved by XPS for Pt/BGTO, and for Pt/BGTO after photocatalytic reaction, Table S3: Comparison of photocatalytic degradation performance of Pt/BGTO and other reported piezo-photocatalysts recently. References [73,74,75,76,77,78,79] are cited in the supplementary materials.
