# Supplementary material for: Piezoelectric Effect Enhanced Photocatalytic Activity of Pt/Bi3.4Gd0.6Ti3O12 Plasmonic Photocatalysis
Source: Nanomaterials (Basel). 2022 Apr 1;12(7):1170. doi: 10.3390/nano12071170 (PMC9000398; doi:10.3390/nano12071170)
Supplement: Supplementary file 1 [file nanomaterials-12-01170-s001.zip › nanomaterials-1637867-SI.pdf]

Supplementary material

Piezoelectric Effect Enhanced Photocatalytic Activity of $Pt/Bi_{3.4}Gd_{0.6}Ti_3O_{12}$ Plasmonic Photocatalysis

Fengjuan Liang, Shijun Wu*, Zhiwu Chen*, and Zhenya Lu

School of Materials Science and Engineering, South China University of Technology, Guangzhou 510641, China; 202021021438@mail.scut.edu.cn (F.L.); zhylu@scut.edu.cn (Z.L.)

* Correspondence: wushijun@scut.edu.cn (S.W.); chenzw@scut.edu.cn (Z.C.)

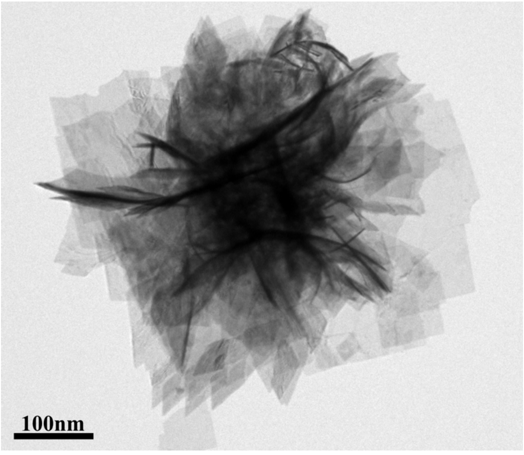

Figure S1. TEM image of synthesized BGTO powders.

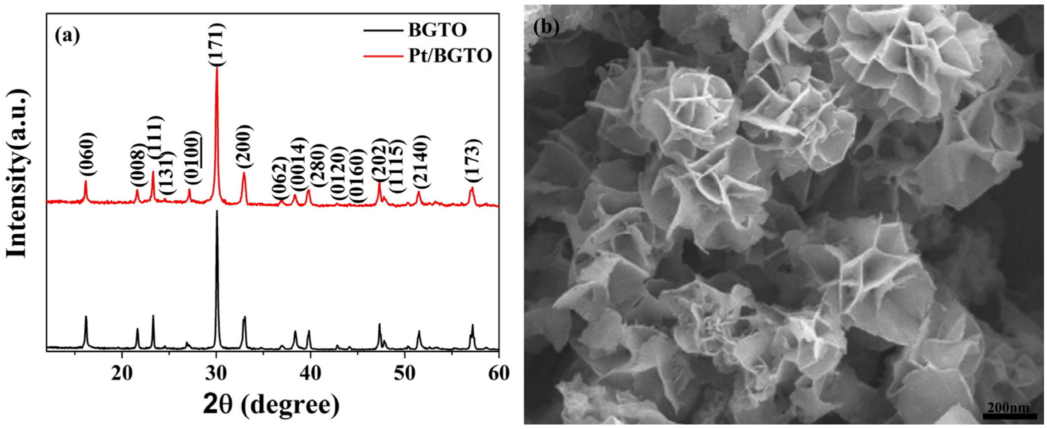

Figure S2. (**a**) XRD pattern, (**b**) SEM image of Pt/BGTO.

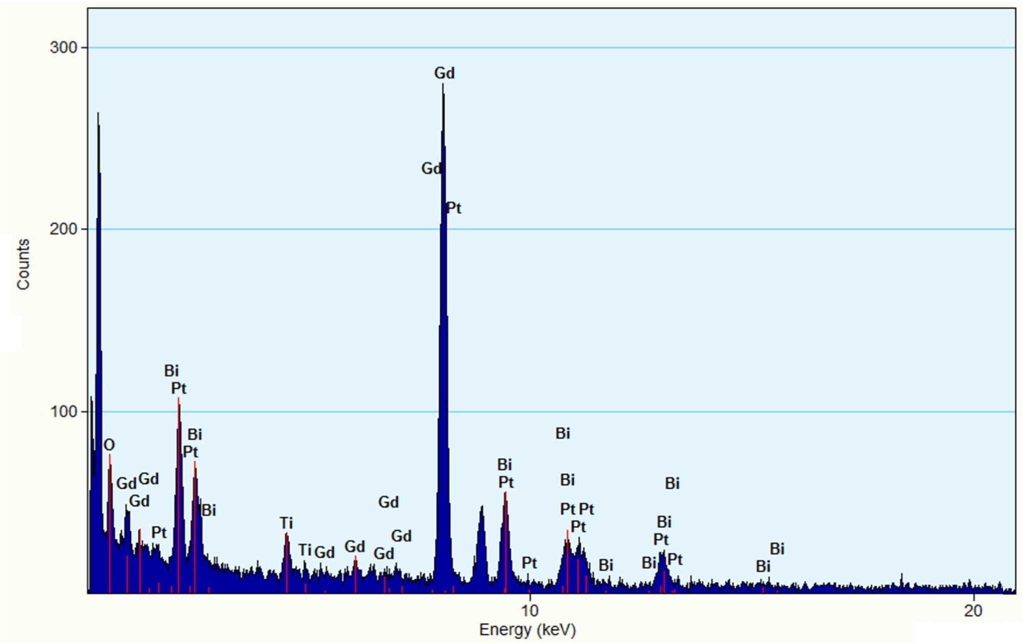

Figure S3. EDS spectrum analysis of the Pt/BGTO.

Table S1 The atomic percentages and weight percentages of Pt Bi, Gd, Ti, O and Pt obtained from the EDS spectrum analysis in Pt/BGTO.

Element	Atomic percentage (%)	Weight percentage of Pt/BGTO (%)
Bi	17.69	60.59
Gd	3.12	8.04
Ti	15.59	12.21
O	62.71	16.43
Pt	0.89	2.73

Table S2 The atomic compositions percentages retrieved by XPS for Pt/BGTO, and for Pt/BGTO after photocatalytic reaction.

Element	Atomic percentage of Pt/BGTO (%)	Atomic percentage of Pt/BGTO after reaction (%)
Bi	17.64	17.66
Gd	3.15	3.16
Ti	15.72	15.74
O	62.56	62.53
Pt	0.93	0.91

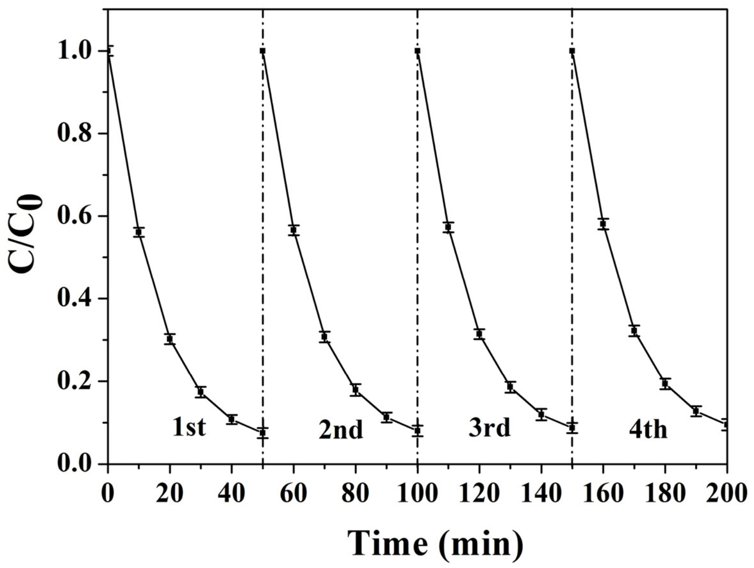

Figure S4. Cycling runs of MO degradation by Pt/BGTO heterojunction under both ultrasonic excitation and whole spectrum light irradiation.

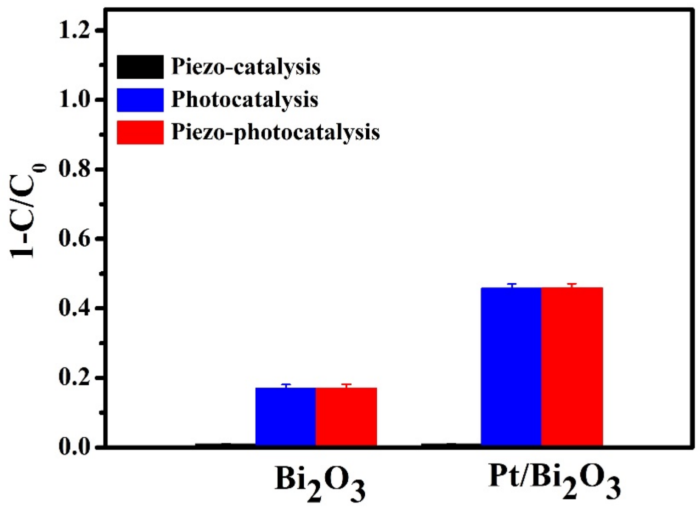

Figure S5. Piezo-catalytic, photocatalytic, and piezo-photocatalytic degradation of MO in the presence of Bi_2O_3 and Pt/Bi_2O_3 samples for 70 min.

We have performed a control experiment depositing PtNPs onto the surface of Bi_2O_3, an inert support (not piezoelectric). The degradation of MO was chosen as the experimental subject to test the photocatalytic performance of Bi_2O_3 and Pt/Bi_2O_3 heterojunction, employing irradiation by light of whole spectrum, ultrasonic excitation as well as both of them together. The comparison of piezo-catalytic, photocatalytic, and piezo-photocatalytic degradation of MO in the presence of Bi_2O_3 and Pt/Bi2O3 samples for 70 min are shown in the following Figure S5. The catalytic process is affected minutely by the ultrasonic excitation alone. Bi_2O_3 and Pt/Bi_2O_3 samples all showed negligible catalytic performance under ultrasonic excitation (the first black column) due to Bi_2O_3 is not piezoelectric material. When we introduced the excitation by whole light spectrum, weak photocatalytic activity was observed for pure Bi_2O_3, with the rate of degradation of MO being less than 17% in 70 min, thus revealing the wide band gap to be the reason behind intrinsic poor photocatalytic effect of Bi_2O_3. In comparison, dramatic enhancement of photocatalytic activity was observed for Pt/ Bi_2O_3 with the consequent degradation of MO being more than 45% within 70 min as a result of the LSPR effect. However, as for both Bi_2O_3 and Pt/Bi_2O_3 samples, no enhancement of the degradation rate of MO was observed when the photo-excitation and ultrasonic excitation were used together. The result of control experiment means that the built-in piezoelectric field in BGTO induced by ultrasonic excitation is the key to promote the mobility of the charge carriers and suppress the recombination of photogenerated carriers, which subsequently contributes to the photocatalysis process.

Table S3. Comparison of photocatalytic degradation performance of Pt/BGTO and other reported piezo-photocatalysts recently

Photocatalyst	Catalytic conditions	Catalyst / Methyl orange	Catalytic efficiency	Ref.
Pt/$Bi_{3.4}Gd_{0.6}Ti_3O_{12}$	Xe lamp (300 W) + ultrasound (53 kHz, 100 W)	20 mg/80 ml (10 mg/L)	92% degrad. eff. within 70 min	This work
$BaZr_{0.02}Ti_{0.98}O_3$	UV light (24 W)+ ultrasound (40 kHz, 70 W)	100 mg/10 ml (6 mg/L)	89% degrad. eff. within 240 min	[73]
Poled Ag/BCT-BZT	Havells lamp (30 W)+ ultrasound (40 kHz, 70 W)	100 mg/10 ml (5 mg/L)	95% degrad. eff. within 90 min	[74]
$BaTiO_3$ nanowires	UV-LED (4.2 W)+ ultrasound (40 kHz, 180 W)	--/100 ml (5 mg/L)	~90% degrad. eff. within 70 min	[75]
MoS_2@Ag_2O	Infrared lamp (300W)+ Ultrasound (--)	20 mg/50 ml (20 mg/L)	100% degrad. eff. within 120 min	[76]
$Bi_{0.5}Na_{0.5}TiO_3$	Xe lamp (300 W)+ ultrasound (28 kHz, 200 W)	10 mg/100 ml (10 mg/L)	55% degrad. eff. within 60 min	[77]
$Na_{0.5}Bi_{2.5}Nb_2O_9$	Xe lamp (300 W)+ ultrasound (40 kHz, 600 W)	50 mg/50 ml (20 mg/L)	~40% degrad. eff. within 120 min	[78]
TiO_2/$PbTiO_3$	UV lamp (30W)+ ultrasound (40 kHz--)	10 mg/100 ml (1 mg/L)	94% degrad. eff. within 120 min	[79]
